# Successful treatment of a pancreatic pseudocyst accompanied by massive hemothorax: a case report

**DOI:** 10.1186/s13256-015-0791-5

**Published:** 2015-12-29

**Authors:** Chiao-Ching Li, Chin-Wen Hsu, Chiao-Zhu Li, Shyh-Ming Kuo, Yu-Chiuan Wu

**Affiliations:** 1 Division of General Surgery, Department of Surgery, Kaohsiung Armed Forces General Hospital, Kaohsiung, Taiwan; 2 Department of Neurological Surgery, Tri-Service General Hospital, National Defense Medical Center, Taipei, Taiwan; 3 Department of Biomedical Engineering, I-Shou University (Yanchao Campus), Kaohsiung, Taiwan; 4 Yuh-Ing Junior College of Health Care and Management, Kaohsiung, Taiwan; 5 National Kaohsiung University of Hospitality and Tourism, Kaohsiung, Taiwan

**Keywords:** Distal pancreatectomy, Hemothorax, Pancreatic pseudocyst

## Abstract

**Background:**

It is rare to encounter massive hemothorax as a complication of pancreatic pseudocyst. In addition, as no obvious hypotension and abdominal discomfort were noted, it was difficult to consider gastrointestinal lesion a possibility.

**Case presentation:**

A 54-year-old Taiwanese man had tightness on the left side of his chest and shortness of breath for 3 days. He had a history of acute pancreatitis 3 months ago. After history taking and a series of examinations including thoracocentesis and computed tomography of his abdomen and chest, the diagnosis was finally confirmed based on the high amylase levels in his pleural fluid.

**Conclusions:**

Treatment with distal pancreatectomy and splenectomy was subsequently successfully performed. Based on our experience, we briefly discuss the currently available treatment options for pancreatic pseudocyst.

## Background

Pleural effusion may result from a variety of causes, including pulmonary, cardiac, hepatic, and renal diseases, with pulmonary conditions being the most common cause. The diagnosis is usually made through comprehensive history taking, physical examination, and a series of workups, including thoracocentesis and imaging studies. However, it is still difficult to diagnose the causes of pleural effusion in many cases and, despite recent efforts aimed at more effectively diagnosing the causes of this condition, approximately 15 to 20 % of all pleural effusion cases remain undiagnosed [1].

In addition, the presence of concomitant intra-abdominal lesions is hard to diagnose in cases of massive pleural effusion without recent abdominal symptoms. In particular, pleural effusion is an uncommon complication of pancreatitis, and massive left-sided hemothorax caused by pancreatic disease is highly rare. In this study, we report on a rare case of massive left-sided hemothorax as a complication of an asymptomatic pancreatic pseudocyst in which, after detailed examination, distal pancreatectomy with splenectomy was successfully performed.

## Case presentation

**Fig. 1:**
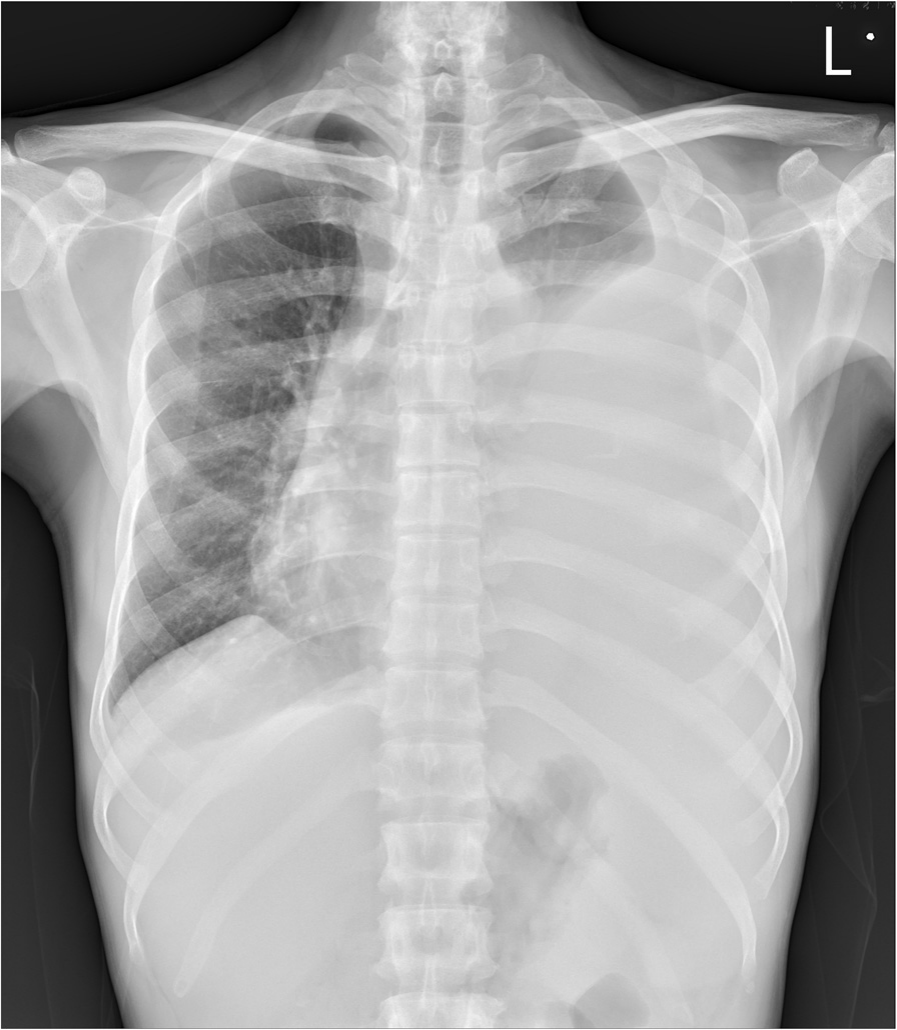
Chest radiograph reveals massive left-sided pleural effusion with a right-deviated trachea

A 54-year-old Taiwanese man visited our cardiovascular department complaining of tightness on the left side of his chest and shortness of breath for 3 days. A chest radiograph revealed massive left-sided pleural effusion with a right-deviated trachea (Fig. 1). He was admitted for further evaluation. He denied a history of cough, hemoptysis, productive sputum, weight loss, abdominal discomfort, or trauma in recent months. At this time, panendoscopy and computed tomography of his abdomen revealed a gastric ulcer and acute pancreatitis with phlegmon formation. After gastrointestinal department follow-up, he reported experiencing symptom relief after 1 month.

On physical examination at admission, his respiratory rate was 24/minute and his blood oxygen saturation (SpO_2_), as measured using a pulse oximeter, was 98 %. Dull percussion and decreased breath sounds were noted in his left thorax. The tentative impression was massive left-sided pleural effusion. Accordingly, we initially performed closed-tube thoracostomy (using a 16-French pigtail catheter), and approximately 1500 mL bloody pleural fluid was drained. Routine blood and urine investigations revealed normocytic anemia without any bleeding tendency. These tests included blood biochemistry tests (renal function, liver function, lipid profile) and electrolyte test. His pleural fluid was bloody, and analysis revealed the following values: protein level, 3.9 g/dL; lactate dehydrogenase, 483 U/L (blood, 160 U/L); glucose, 114 mg/dL; white blood cell count, 900/μL; red blood cell count, 54,000/μL; and hematocrit, 3.7 % (blood, 28.1 %). According to Light’s criteria, the patient’s pleural fluid was exudative, and some mesothelial and neutrophilic cells were found in the pleural effusion cytology. However, the subsequent culture was sterile.

**Fig. 2:**
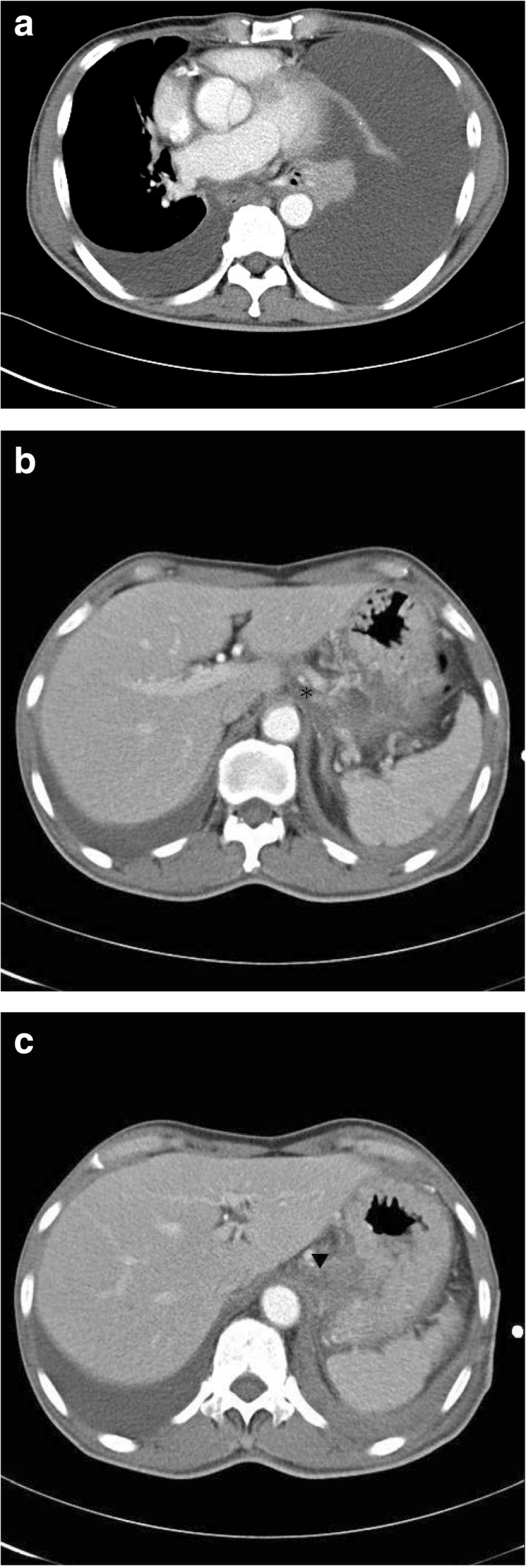
Computed tomography scans of the chest and abdomen. **a** The computed tomography scan of the chest reveals left massive hydropneumothorax with collapse of most of the left lung and shifting of the mediastinum to the right side. **b** The *asterisk* shows the pseudocyst in the pancreatic tail. **c** The *inverted triangle* shows the pseudocyst in the pancreatic tail

In addition, all tumor markers were within the normal ranges: prostate-specific antigen, 0.847 ng/mL; alpha-fetoprotein, 2.47 ng/mL; carbohydrate antigen 19-9, 3.58 U/mL; carcinoembryonic antigen, 0.97 ng/mL. Moreover, echocardiography was performed to rule out cardiogenic disease, and revealed normal chamber size and wall thickness, although akinesis of his left ventricle, inferiorly, posteriorly, and on the lateral wall, was noted. Furthermore, hypokinesis of the anterior and septal wall with severe left ventricle systolic dysfunction was observed. A chest computed tomography subsequently revealed left massive hydropneumothorax with collapse of most of his left lung and shifting of his mediastinum to the right side, as well as pleural effusion in his right lung with subsegmental atelectasis of the right lower lobe of his lung; however, no cardiovascular abnormality was detected (Fig. 2a).

Owing to his history of acute pancreatitis, computed tomography of his abdomen was also performed based on the gastroenterologist’s suggestion. The computed tomography scan revealed a pseudocyst in his pancreatic tail and indicated a diverticulum in the lower third of his esophagus (Fig. 2b and c).

Based on these findings, further examinations were arranged, including panendoscopy, esophagography, aortography, and bronchial arteriography. However, all findings were unremarkable. Moreover, obstruction of the pigtail catheter was noted on the left side of his chest wall, and right closed-tube thoracostomy was performed because of pleural effusion in his right lung. This time, the amylase and lipase levels in his pleural fluid were analyzed. The pleural fluid amylase level was found to be increased to 30,431 U/L. Accordingly, we suspected that his massive left-sided hemothorax was related to the pancreatic pseudocyst, and a general surgeon was consulted.

**Fig. 3:**
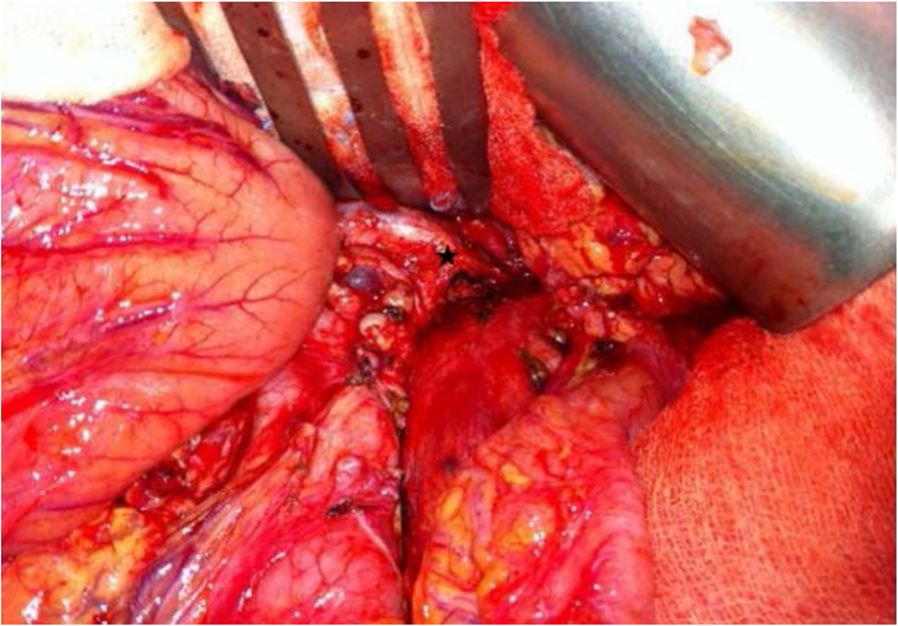
Distal pancreatectomy and splenectomy. The pseudocyst is seen in the pancreatic tail (*star*)

In consequence, the patient underwent distal pancreatectomy and splenectomy. Fistula formation was observed in his periesophageal region, and pseudocyst formation was found at his pancreatic tail, as well as at the lesser sac (Fig. 3). The total surgical time was approximately 5 hours. Drainages were placed from Morison’s pouch and the spleen fossa. Post-surgery, his hemothorax recovered and his general condition consequently improved; he was discharged 2 weeks after the operation without the use of a somatostatin analogue (octreotide).

## Discussion

The etiologies of pleural effusion range from cardiopulmonary diseases to symptomatic inflammatory or malignant diseases; in addition, ascites or lesser sac fluid collection in chronic pancreatic disease may also be associated with pleural effusion. While tube thoracostomy drainage can be used to identify and diagnose hemothorax, pancreatic disease is generally not considered in patients solely presenting with massive hemothorax [2]. Thus, it is important that any history of pancreatitis is noted, as one of the complications of non-traumatic pancreatitis is pancreatic pseudocysts. In general, pancreatic pseudocysts develop approximately 3 to 4 weeks after acute pancreatitis, and are most often manifested by an epigastric mass or sensation of fullness. Moreover, bleeding, fistula formation, infection, and extension are known complications of pseudocysts [3].

In our case, we considered the hemothorax to be related to the pancreatic pseudocyst, and the diagnosis was confirmed by very high amylase levels in our patient’s pleural fluid and by the abdominal computed tomography findings. Enzyme-rich pleural effusions represent a disruption of the dorsal pancreatic ductal system [4]; therefore, this fluid leakage must be stopped.

Decisions on the treatment of pancreatic pseudocysts are currently based on the clinical setting, the presence or absence of symptoms, the stage and size of the pseudocyst, and the presence or absence of complications [4]. The currently available treatment options include observation, percutaneous catheter drainage, endoscopic drainage, and surgical intervention [5]. Observation is generally only performed for asymptomatic pseudocysts that remain stable or diminish in size [6]; however, attention should be paid to the occurrence of complications such as infection, fistula, intractable pain, or rupture [7, 8]. Percutaneous catheter drainage is generally indicated for pseudocysts only after a 6-week delay to allow the pseudocyst wall to thicken and mature [9]. Its contraindications include suspicion of malignancy, collections associated with a solid or non-drainable pancreatic mass, lack of a safe access route, recent or active hemorrhage into the collection (the presence of an arterial pseudoaneurysm), and collections associated with obstruction of the main pancreatic duct [10, 11]. Furthermore, endoscopic drainage of pancreatic pseudocysts appears to be a safe, effective, and definitive treatment for patients in whom anatomic considerations allow its use [12]. One such endoscopic method, endoscopic retrograde cholangiopancreatography, can be used to open the sphincters of the pancreatic valve ducts, thereby resulting in decompression of the pancreatic fluid collection, and can, moreover, aid in the stent placement in cases of pancreatic ductal disruption [4]. The drawbacks of endoscopic methods include the need for extensive training and experience of the gastroenterologist, the need for a surgical backup plan, as well as the fact that the presence of an abnormal pancreatic duct system makes this procedure difficult to perform. However, surgery remains the standard method for drainage of pancreatic pseudocysts, against which new methods are being compared. The surgical treatment options consist of gastrocystostomy, duodenocystostomy, Roux-en-Y cystojejunostomy, and distal pancreatectomy (left-sided resection), depending on the leakage lesion. However, these operative procedures reportedly carry a 10 to 30 % morbidity rate, a 1 to 5 % mortality rate, and a 10 to 20 % recurrence rate [13]. Gastrocystostomy and jejunocystostomy are alternative procedures for establishing internal decompression [14], and duodenocystostomy is occasionally indicated for small cysts in the pancreatic head [15], whereas distal pancreatectomy is indicated for pancreatic lesions extending to the left of the midline and that do not include the duodenum and distal bile duct.

In addition, atypical presentations of pancreatic pseudocysts are reported, such as mediastinal pancreatic pseudocyst [16] or a cyst mimicking neoplasm of the pancreas [17].

The differential diagnosis needs to be determined carefully. Finally, we need to consider that intra-abdominal pressure is related to pancreatic pseudocysts [18]. If a patient has a history of pancreatitis, we should measure the intra-abdominal pressure if a pseudocyst is detected. Pancreatic pseudocyst is induced by abdominal hypertension and abdominal compartment syndrome.

## Conclusions

In our case, the pancreatic pseudocyst was located at the pancreatic tail and below the lesser sac, and fistula formation was observed from the peripancreatic to the periesophageal region. We did not consider percutaneous catheter drainage because of the presentation of hemothorax; instead, distal pancreatectomy with splenectomy was performed, and the hemothorax was consequently successfully treated. Based on this case, we believe that this treatment regimen should be considered in similar cases.

## Consent

Written informed consent was obtained from the patient for publication of this case report and any accompanying images. A copy of the written consent is available for review by the Editor-in-Chief of this journal.

## References

[1] Hirsch A, Ruffle P, Nebut M, Bignon J, Chrétien J. Pleural effusion-laboratory tests in 300 cases. Thorax. 1979;34:106–12. 10.1136/thx.34.1.106 PMC471017 441988

[2] Tewari SC, Jayaswal R, Chauhan MS, Kaul SK, Narayanan VA. Bilateral recurrent haemorrhagic pleural effusion in asymptomatic chronic pancreatitis. Thorax. 1984;44:824–5. 10.1136/thx.44.10.824 PMC1020852 2595625

[3] Cochran JW. Pancreatic pseudocyst presenting as massive haemothorax. Am J Gastroenterology. 1978;69:84–7. 645692

[4] Traverso LW, Kozarek RA. Interventional management of pancreatic fluid collections. Surg Clin North Am. 1999;79:745–57. 10.1016/s0039-6109(05)70040-2 10470324

[5] Andren-Sandberg A, Ansorge C, Eiriksson K, Glomsaker T, Maleckas A. Treatment of pancreatic pseudocysts. Scand J Surg. 2005;94:165–75. 10.1177/145749690509400214 16111100

[6] Bourliere M, Sarles H. Pancreatic cysts and pseudocysts associated with acute and chronic pancreatitis. Dig Dis Sci. 1989;34:343–8. 10.1007/BF01536253 2646086

[7] Vitas GJ, Sarr MG. Selected management of pancreatic pseudocyst: operative versus expectant management. Surgery. 1992;111:123–30. 1736380

[8] Yeo CJ, Bastidas JA, Lynch-Nyhan A, Fishman EK, Zinner MJ, Cameron JL. The natural history of pancreatic pseudocysts documented by computed tomography. Surg Gyncecol Obstet. 1990;170:411–17. 2326721

[9] Martin EW, Catalano P, Cooperman M, Hecht C, Carey LC. Surgical decision-making in the treatment of pancreatic pseudocysts. Internal versus external drainage. Am J Surg. 1979;138:821–4. 10.1016/0002-9610(79)90304-0 507299

[10] Pitchmumoni CS, Agarwal N. Pancreatic pseudocysts. When and how should drainage be performed? Gastroenterol Clin North Am. 1999;28:615–39. 10.1016/s0889-8553(05)70077-7 10503140

[11] Nealon WH, Walser E. Main pancreatic ductal anatomy can direct choice of modality for treating pancreatic pseudocysts (surgery versus percutaneous drainage). Ann Surg. 2002;235:751–8. 10.1097/00000658-200206000-00001 PMC1422503 12035030

[12] Schutz SM, Keung JW. Pancreatic endotherapy for pseudocysts and fluid collections. Gastrointest Endosc. 2002;56:150–2. 10.1067/mge.2002.125232 12085059

[13] Williams KJ, Fabian TC. Pancreatic pseudocysts: recommendations for operative and nonoperative management. Am Surg. 1992;58:199–205. 1558338

[14] Frey CF. Pancreatic pseudocyst-operative strategy. Ann Surg. 1978;188:652–62. 10.1097/00000658-197811000-00012 PMC1396778 309751

[15] Schattenkerk ME, De Vries JE, Bruining HA, Eggink WF, Obertop H. Surgical treatment of pancreatic pseudocysts. Br J Surg. 1972;123:209–21. 10.1002/bjs.1800691012 7127039

[16] Gupta R, Munoz JC, Garg P, Masri G, Nahman Jr NS, Lambiase LR. Mediastinal pancreatic pseudocyst-a case report and review of the literature. MedGenMed. 2007;9:8. PMC1994836 17955064

[17] Gintowt A, Hac S, Dobrowolski S, Sledziński Z. An unusual presentation of pancreatic pseudocyst mimicking cystic neoplasm of the pancreas: a case report. Cases J. 2009;2:9138. 10.1186/1757-1626-2-9138 PMC2803935 20062655

[18] Papavramidis TS, Duros V, Michalopoulos A, Papadopoulos VN, Paramythiotis D, Harlaftis N. Intra-abdominal pressure alterations after large pancreatic pseudocyst transcutaneous drainage. BMC Gastroenterol. 2009;9:42. 10.1186/1471-230X-9-42 PMC2700125 19500396

